# Thermal Wave Scattering by an Elliptic Subsurface Hole Buried in a Block, Based on the Non-Fourier Equation

**DOI:** 10.3390/s19081878

**Published:** 2019-04-19

**Authors:** Chuanping Zhou, Ban Wang

**Affiliations:** 1 College of Electrical Engineering, Zhejiang University; Hangzhou 310027, China; zhouchuanping@126.com; 2 School of Mechanical Engineering, Hangzhou Dianzi University; Hangzhou 310018, China

**Keywords:** non-Fourier law, two boundaries, elliptic hole, thermal wave scattering, complex function method

## Abstract

With the application to engineering practice, the study of the scattering of thermal waves using innovative and comprehensive methods is becoming increasingly important. The thermal wave scattering by an elliptic subsurface hole in a block with two boundaries is discussed based on the non-Fourier heat conduction equation, employing the complex function method and the conformal mapping method, and a general solution for the thermal wave scattering is given. The numerical results of temperature distributions around a subsurface hole are presented and the effects of geometrical and physical parameters on the temperature distributions were analyzed. The wave number, the shape and position of the hole, the scale of the block, and the frequency of the heat load were found to have great effects on distributions and variations of temperature. The findings of this study could be helpful in providing better understandings of infrared thermal wave imaging, the physical inverse problem, and the evaluation of internal holes in materials.

## 1. Introduction

Subsurface defects can be detected and evaluated by tuning the temperature in the thermal wave field, since the frequency and amplitude of ultra-short laser pulses can be controlled [1,2,3]. It is an accessible way to get the subsurface micro-structure information and to realize thermal wave detection according to the real-time measurement of the temperature field on the solid surface. When the temperature increases quickly, the heat conduction process in solids should be described by a hyperbolic equation, which means that only wave equations can be used to illustrate the features and properties of the heat conduction process [4,5,6]. It has been proven that results calculated based on the non-Fourier equation are more consistent with engineering practices [7,8,9]. In most cases, the classical Fourier heat conduction law is an excellent description of heat conduction physics. In practical engineering, heat sources such as lasers and microwaves with extremely short durations, very high frequencies, or quite high heat-flux densities are widely used. The non-Fourier heat conduction phenomenon has been found in these media. Many researchers have attached much importance to the potential practical values of non-Fourier heat conduction in many applications, and non-Fourier heat conduction has become one of the hotspots in the field of heat transfer.

It can also evaluate the applicable conditions for the classic heat conduction equation, which has great prospects in theory and engineering for analyzing the thermal wave multiple scattering and temperature distribution with the hyperbolic heat conduction equation.

Most recently, with the advantages of non-contact accuracy and sensitivity, infrared thermography is frequently used in the nondestructive evaluation of solid materials containing subsurface inclusions [3,5,10]. Thermal wave detection technology using the infrared lock-in thermography method adopts the heat source of the single–frequency modulation intensity of the sine law [11]. The scattering of thermal waves can also have applications in other fields, such as thermal recovery from heavy oil reservoirs [12,13] and the sensing property of defective temperature sensors. Consequently, investigations into the scattering of thermal waves are becoming increasingly important. Nevertheless, most studies on the scattering of thermal waves are aimed at infinite body models or semi-infinite body models with a single structure boundary [14,15,16]. These models are usually applied to the structures in which the scale along the thermal perturbation propagation direction is much larger than the thermodynamics thickness [17]. Besides, the shapes of scattered bodies are almost always assumed to be circular and the effects resulting from the finiteness of other scales in project practice are also ignored [16,18]. This means that the applications of the theories outlined above have limitations in engineering practice. Therefore, it is necessary to pursue innovative and comprehensive methods.

The main objective of this paper was to investigate the multiple scattering of thermal waves by a subsurface, non-circular hole in an infinite block with two boundaries, based on the non-Fourier heat conduction hyperbolic equation. The problem can be reduced to the solution of an infinite system of algebraic equations. Compared to previous theoretical study, the outstanding novelties of this study are given as follows. The non-Fourier heat conduction equation can be used to calculate the temperature and thermal stress of materials and structures in extreme environments, but the traditional Fourier equation is not invalid. The Fourier equation is applicable in an infinite body, but the calculation error is larger in structures with boundaries. Our theory can be used to calculate the thermal wave scattering around non-circular subsurface holes. First, the wave equation of heat conduction and its general solution are described. Then, as examples, numerical results of thermal wave scattering in the block with an elliptical hole are computed. Lastly, the effects of incident wave number, physical dimensions, and parameters on the temperature distribution are analyzed, especially those of the incident wave number. Given proper mapping functions, this paper brings about an important significance in solving the problem of the scattering of thermal waves by holes in any shapes [19].

## 2. Wave Equation of Heat Conduction and Its General Solution

**Figure 1:**
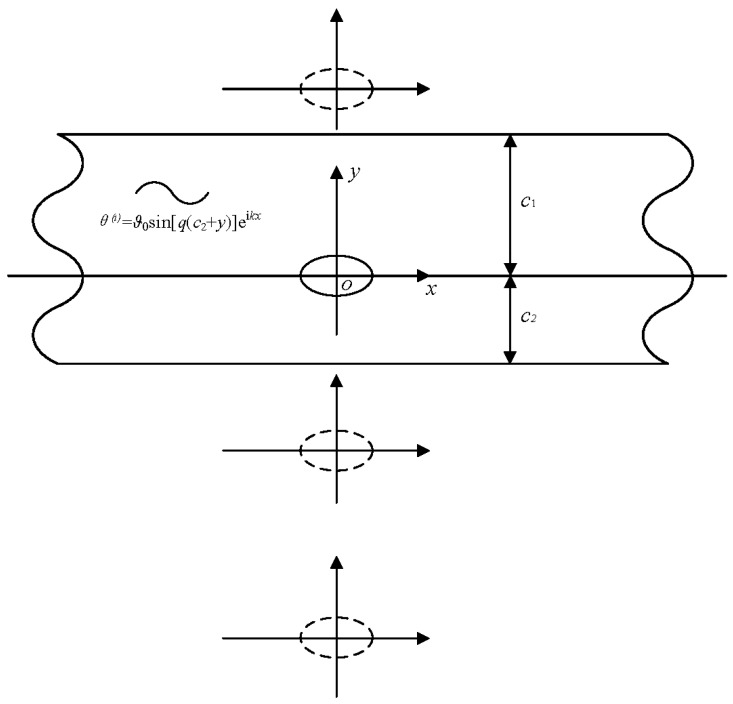
Laser pulse heating on specimen.

An infinite block with a hole was considered, and is depicted in Figure 1. An elliptic hole was embedded in an adiabatic solid. An ultra-short laser pulse, modulated at a frequency *f*, hit the surface of the heated materials along the *z* direction. Providing the temperature field distribution along the *z* direction is homogeneous, the temperature is determined by *x* and *y* only.

Based on the non-Fourier law of heat conduction, the governing equation of temperature in the solid can be written as:

(1) $$\nabla^{2}T=\frac{1}{c^{2}}\frac{\partial^{2}T}{\partial t^{2}}+\frac{1}{D}\frac{\partial T}{\partial t},$$

where $\nabla^{2}$ is the Laplace operator, and $\nabla^{2}=\frac{\partial^{2}}{\partial x^{2}}+\frac{\partial^{2}}{\partial y^{2}}$; λ, $c_{p}$, and ρ are the thermal conductivity, the specific heat at constant pressure, and the density, respectively. The thermal diffusivity is $D=\lambda /\rho c_{p}$, $c=\sqrt{D/\tau }$ is the thermal wave propagation velocity, τ is the heat relaxation time, and T is the temperature of the solid medium.

This divides the solution of a wave propagation problem into both a time domain solution and a frequency domain solution. By means of the Fourier transform, the two kinds of solutions can be transformed. To detect the position, direction, and size of flaws in the materials, employing the phase and amplitude of thermal response, as well as wave number and the space attenuation coefficient, periodic thermal waves are excited in the materials.

According to the Fourier decomposition theorem, the periodic heat conduction process is considered to be the composition of several simple harmonic waves. Periodic steady conduction can be analyzed in light of the format standardization of wave theory.

As the solution to Equation (1) is investigated, the temperature field can be described as

(2) T=Tm+Re[ϑexp(−iω t)]

where ϑ is the temperature amplitude, which should meet with the Helmholtz equation as follows:

(3) $$\nabla^{2}\vartheta +\kappa^{2}\vartheta =0$$

In which Re denote real part; $T_{m}$ is the ambient mean temperature; ω is the incident frequency, ω=2πf; $i=\sqrt{-1}$ is the imaginary unit; κ is the wave number of complex variables, $\kappa ={\left(\frac{\omega^{2}}{c^{2}}+i\frac{\omega }{D}\right)}^{1/2}=\alpha +i\beta$; and α, β are the wave number and the absorption coefficient of thermal waves, respectively. Without loss of generality, after normalizing and taking α<0, β<0, we can obtain

$$\alpha =\sqrt{\frac{1}{2}[\sqrt{\frac{\omega^{4}}{c^{4}}+\frac{\omega^{2}}{D^{2}}}+\frac{\omega^{2}}{c^{2}}]}=\sqrt{\sqrt{\frac{1}{4}k^{4}+\frac{1}{\mu^{4}}}+\frac{1}{2}k^{2}}$$

$$\beta =\sqrt{\frac{1}{2}[\sqrt{\frac{\omega^{4}}{c^{4}}+\frac{\omega^{2}}{D^{2}}}-\frac{\omega^{2}}{c^{2}}]}=\sqrt{\sqrt{\frac{1}{4}k^{4}+\frac{1}{\mu^{4}}}-\frac{1}{2}k^{2}}$$

Here, *k* is the wave number of thermal waves without diffusive effect. When the propagating speed of the thermal wave is c→∞, the non-Fourier equation degenerates into the classical Fourier equation, and then

$$\alpha \to \sqrt{\omega /2D}=\sqrt{\pi f/D}=1/\mu ,\;\beta \to \sqrt{\omega /2D}=1/\mu$$

while $\kappa =\alpha +i\beta \to (1+i)\sqrt{\omega /2D}=\left(1+i\right)/\mu$.

Consequently, there exists the wave motion with the form $\vartheta e^{-i\omega t}=Ae^{-\beta x}e^{i(\alpha x-\omega t)}$, which denotes the propagating thermal waves with their vibration amplitude attenuating in the *z* direction.

Here, a complex function method is employed, with the complex variable

(4) $$z=x+iy,\;\bar{z}=x-iy;$$

that is,

(5) $$x=\frac{1}{2}(z+\bar{z}),\;y=\frac{1}{2i}(z-\bar{z}).$$

In this way, Equation (3) is converted into the form:

(6) $$\frac{\partial^{2}\vartheta }{\partial z\partial \bar{z}}+{\left(\frac{\kappa }{2}\right)}^{2}\vartheta =0.$$

The general solution to the scattered field of thermal waves in the solid medium determined by Equation (6) can be described as

(7) $$\vartheta =\sum_{n=-\infty }^{\infty }A_{n}H_{n}^{\left(1\right)}\left(\kappa r\right)e^{in\theta }=\sum_{n=-\infty }^{\infty }A_{n}H_{n}^{\left(1\right)}\left(\kappa \left|z\right|\right){\left\{\frac{z}{\left|z\right|}\right\}}^{n},$$

where $A_{n}$ are the mode coefficients resulting from the subsurface cylinder hole and are determined by the boundary conditions, and $H_{n}^{\left(1\right)}(\cdot )$ is the Hankel function of the first kind.

## 3. The General Solution for the Scattering of Thermal Waves by Subsurface Holes Buried in an Infinite Block

In the process of solving the problem of the scattering of thermal waves by elliptically shaped holes, the conformal mapping method is a feasible way to provide access to meet with the boundary conditions of the hole. The boundaries of non-circular holes in the z plane can be mapped into a unit circle in the ζ plane by using the following equation:

(8) z=Ω(ζ).

As the polar coordinates system is mentioned, there is $z=re^{i\varphi }$ in the z plane, while $\zeta =\rho e^{i\theta }$ is in the ζ plane. In this way, the general solution to the scattered field of thermal waves by non-circular holes in the ζ plane can be described as:

(9) $$\vartheta =\sum_{n=-\infty }^{\infty }A_{n}H_{n}^{\left(1\right)}\left(\kappa \left|\Omega (\zeta )\right|\right){\left\{\frac{\Omega (\zeta )}{\left|\Omega (\zeta )\right|}\right\}}^{n}.$$

For an ellipse with a major radius $r_{1}$ and a minor radius $r_{2}$, the conformal mapping function is denoted as:

(10) $$z=\Omega (\zeta )=\frac{a}{1+\varepsilon }\left(\zeta +\frac{\varepsilon }{\zeta }\right),$$

where $a=\left(r_{1}+r_{2}\right)/2,\varepsilon =\left(r_{1}-r_{2}\right)/\left(r_{1}+r_{2}\right).$

## 4. The Incidence of Thermal Waves and Total Wave Field

Thermal waves can be generated at the fore end of the block by a laser beam with a modulated ultra-short pulse. It propagates along the positive *x* direction. Based on the interference theory of wave field, the temperature distribution in the infinite block is described as:

(11) ϑ=f(y)exp[i(px−ωt)],

which needs to meet with Equation (2); then, Equation (12) is accessible as:

(12) f(y)=Acos(q y)+Bsin(qy),

where p and q are longitudinal wave number and lateral wave number, respectively, and $p^{2}=\kappa^{2}-q^{2}$.

Meanwhile, the upper and lower signs refer to

(13) $$f(c_{1})\exp\left(ipx\right)=0;\;f(-c_{2})\exp\left(ipx\right)=0.$$

In this way, the lateral wave number is derived:

(14) $$q=\frac{n\pi }{c_{1}+c_{2}}\;(n=0,1,2\cdots \infty )$$

Substituting Equation (14) into Equation (6), the following equation is obtained:

(15) $$\vartheta =B\frac{\sin\left[q\left(c_{2}+y\right)\right]}{\cos\left(qc_{2}\right)}\exp\left[i\left(px-\omega t\right)\right].$$

The thermal wave can be described as:

(16) $$\vartheta^{\left(i\right)}=\vartheta_{0}\sin\left[q\left(c_{2}+y\right)\right]e^{ipx-\omega t}=\vartheta_{0}\sin\left[q\left(c_{2}+y\right)\right]\sum_{n=-\infty }^{\infty }i^{n}J_{n}\left(pr\right)e^{in\theta }e^{-i\omega t},$$

where $\vartheta_{0}$ is the temperature amplitude of the incidence thermal wave, that is, excess temperature $\vartheta_{0}=T_{0}-T_{m}$; $q=\pi /\left(c_{1}+c_{2}\right)$, p is the wave number along the *x* direction, and $J_{n}(\cdot )$ is the Bessel function.

Correspondingly, under the adiabatic conditions, the thermal wave can be described as:

(17) $$\vartheta^{\left(i\right)}=\vartheta_{0}\cos\left[q\left(c_{2}+y\right)\right]e^{ipx}e^{-i\omega t}.$$

Taking the multiple scattering by the solid boundary when $y=c_{1}$ and $y=-c_{2}$ ($c_{1}<0,c_{2}<0$) into consideration, the scattered field of thermal waves by non-circular holes can be described in the polar coordinates system as:

(18) $$\vartheta^{\left(s\right)}=\sum_{n=-\infty }^{\infty }A_{n}H_{n}^{\left(1\right)}\left(\kappa r\right)e^{in\varphi }+\sum_{m=1}^{\infty }\sum_{n=-\infty }^{\infty }A_{n}H_{n}^{\left(1\right)}\left(\kappa r_{m}\right)e^{in\varphi_{m}}.$$

By means of the complex function method, we can obtain the complex function expression of Equation (18) in Appendix A.

Consequently, the total wave field is composed of the incidence and scattering fields, which is conveyed by Equation (20):

(19) $$\vartheta^{\left(t\right)}=\vartheta^{\left(i\right)}+\vartheta^{\left(s\right)}.$$

## 5. Mode Coefficient of Waves and Temperature Distribution

Substituting Equation (19) into the Dirichlet boundary condition of heat conduction, the following equation is obtained:

(20) $$\sum_{n=-\infty }^{+\infty }{Ε}_{n}X_{n}=Ε,$$

the expression of which is shown in Appendix B.

When Equation (20) is multiplied by $e^{-is\theta }$ at both ends, and by evaluating its integrals from −π to π, the infinite algebraic equation is described as:

(21) $$\sum_{n=-\infty }^{+\infty }{Ε}_{ns}X_{n}={Ε}_{s}\;(n=s=0,\;\pm 1,\;\pm 2\cdots ).$$

Equation (21) is the infinite algebraic equation determining the mode coefficients of thermal waves.

Let the major radius $r_{1}$ of the subsurface ellipse hole be the characteristic length, and the amplitude of the incident temperature be $\left|\vartheta_{0}\right|$. During computation, the following dimensionless variables were adopted: the ratios between the major radius and the minor radius of ellipse hole were $r_{1}/r_{2}=3/4,1.0,4/3$, respectively. The wave number of the non-diffusive propagating waves was ka=0.01−2.0, the relative length of thermal diffusion was μ/a=0.1−5.0, the distance from the center of the ellipse to the upper and lower signs of the block was $c_{2}/a=5.0-10.0$, $c_{1}/a=5.0-30.0$, and the ratio of the excess temperature was $\vartheta /\vartheta_{0}$.

Thus, the expression of the temperature distribution at the surface is written as:

(22) $$\begin{array}{c} \vartheta =\vartheta_{0}\sin\left[q\left(c_{2}+y\right)\right]e^{ip(x+b)}+\sum_{n=-\infty }^{\infty }A_{n}H_{n}^{\left(1\right)}\left(\kappa \left|z\right|\right){\left\{\frac{z}{\left|z\right|}\right\}}^{n}\\ +\sum_{n=-\infty }^{\infty }A_{n}\sum_{l=1}^{4}\sum_{m=1}^{\infty }{\left(-1\right)}^{l}[H_{n}^{\left(1\right)}\left(\kappa \left|z-z_{lm}\right|\right){\left\{\frac{z-z_{lm}}{\left|z-z_{lm}\right|}\right\}}^{{\left(-1\right)}^{l}n} \end{array}$$

## 6. Numerical Examples

**Figure 2:**
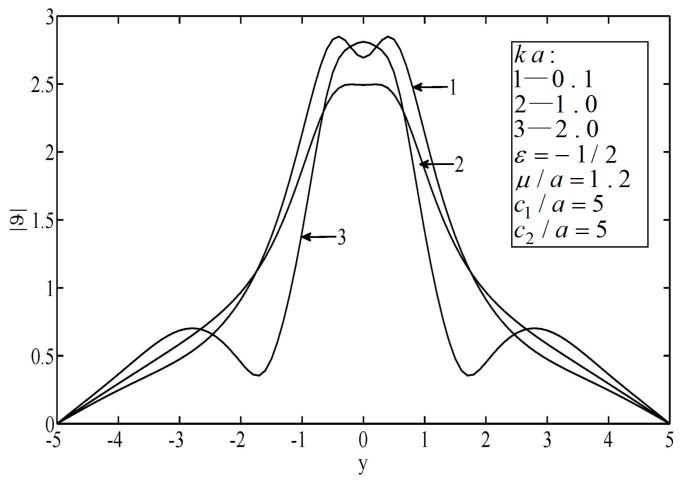
Temperature on the surfaces of the block.

**Figure 3:**
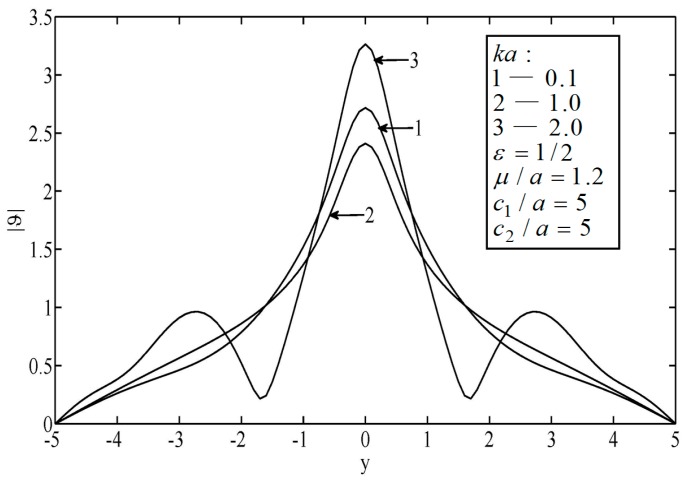
Temperature on the surfaces of the block.

Figure 2 and Figure 3 illustrate the temperature distributions of thermal scattering by a subsurface hole in an infinite block. It can be seen that the peak temperature on the surface of the material appeared right ahead of the elliptical hole. Note that when the wave number of the non-diffusive incident wave was small (e.g., ka<0.3), the wave characteristics of heat conduction were weak. In this case, the temperature distribution was approximately the same as in the results from the classical heat conduction equation, which implies the applicability of the classical heat conduction equation. Furthermore, it was observed that the temperature varied slowly when the wave number was small (e.g., *ka* = 0.1). Otherwise, the temperature varied drastically.

**Figure 4:**
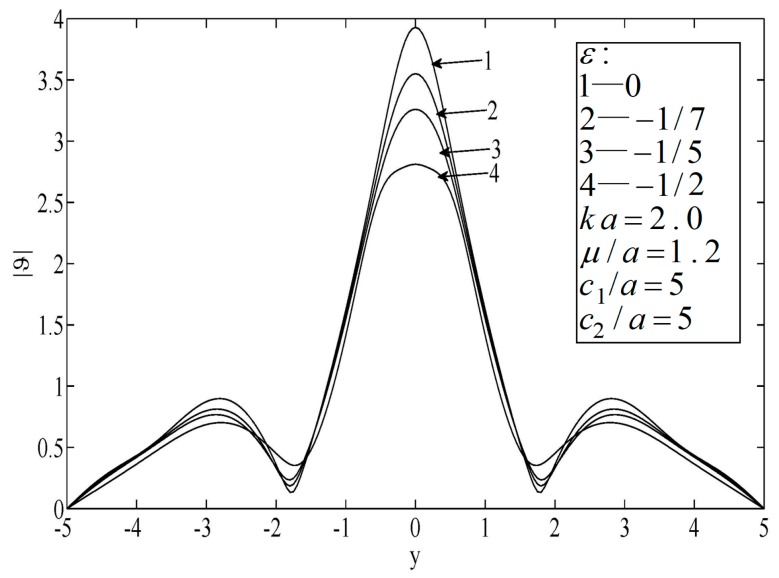
Temperature on the surfaces of the block.

**Figure 5:**
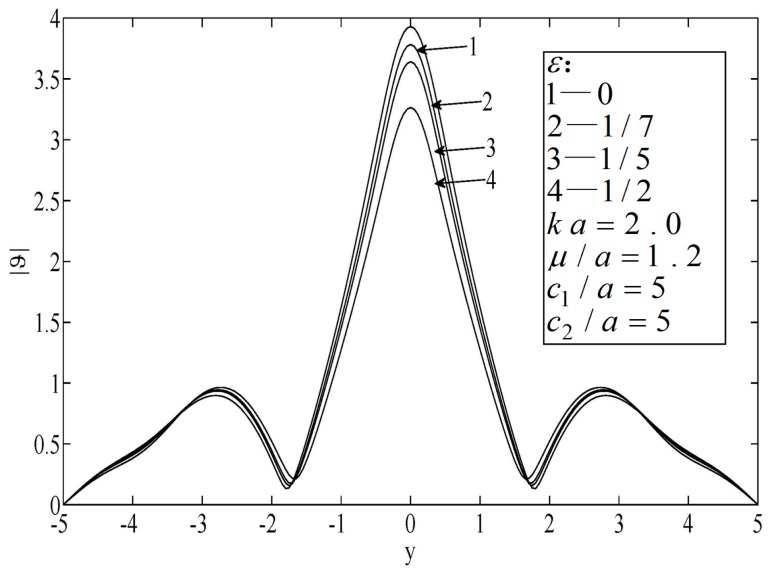
Temperature on the surfaces of the block.

Figure 4 and Figure 5 illustrate the temperature distributions of thermal scattering corresponding to different eccentricities of the ellipse. Specifically, ε=0, |ε|=1/7, and |ε|=1/3 represent the cases of a circular hole, a small eccentricity ratio, and a large eccentricity ratio, respectively. It is shown that the temperature corresponding to the case of a circular hole was the highest among the three at any point. In addition, when the major radius of the ellipse was along the lateral scale (i.e., ε<0), the temperature fluctuated mildly and its peak value dropped slowly, and vice versa (ε<0). Furthermore, only when the center of the hole was on the median of the block was the temperature distribution symmetrical.

**Figure 6:**
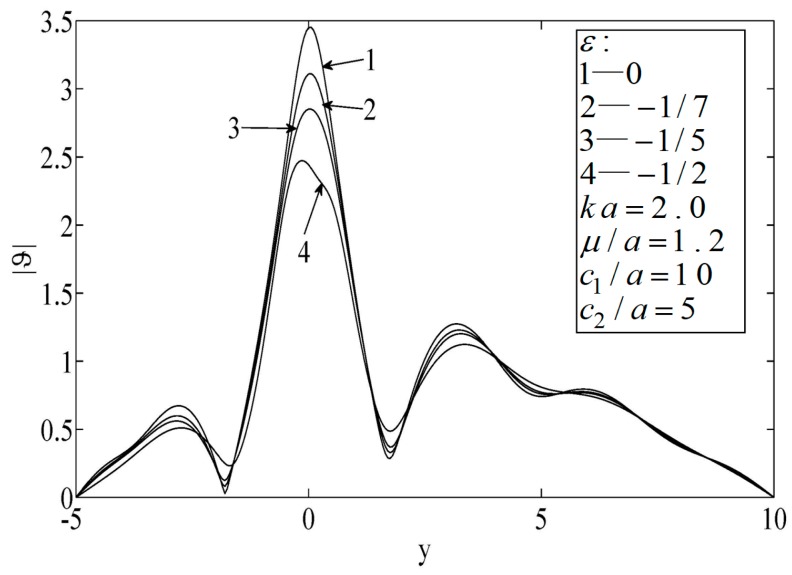
Temperature on the surfaces of the block.

**Figure 7:**
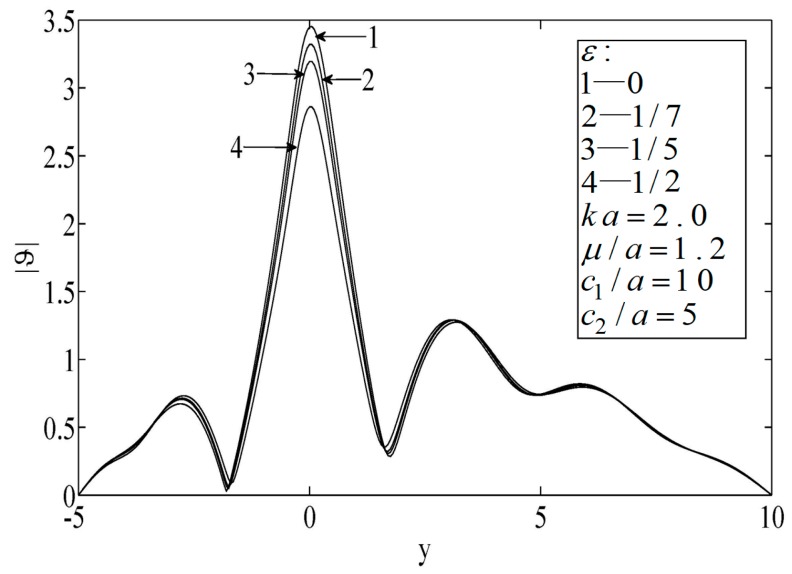
Temperature on the surfaces of the block.

**Figure 8:**
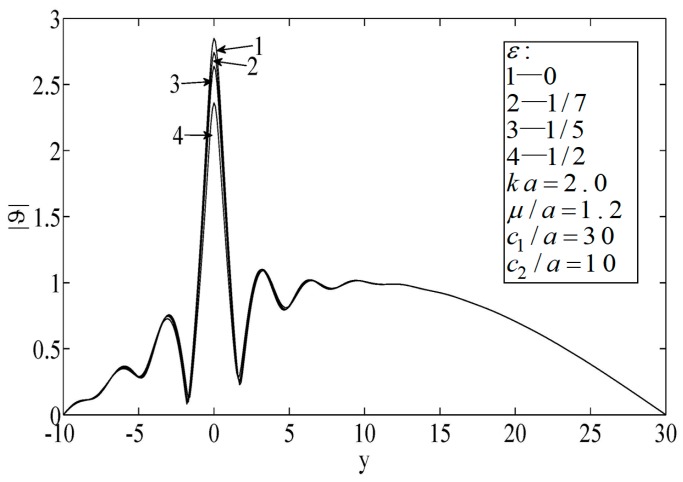
Temperature on the surfaces of the block.

As shown in Figure 6, Figure 7 and Figure 8, the peak temperature appeared at the median of the block when the hole was eccentric to the block, which indicates that the temperature directly in front of the hole decreased. When the size of the block was small, the peak temperature on the surface was relatively high.

**Figure 9:**
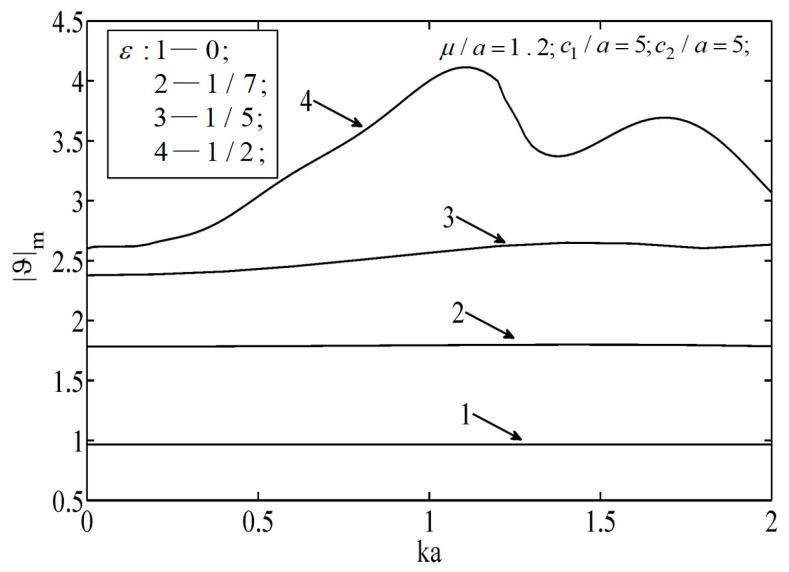
Temperature on the surfaces of the block.

Finally, according to Figure 9, a large wave number resulted in a high-frequency vibration (i.e., a short wave case), in which case the thermal wave propagation exhibited the properties of microparticles.

## 7. Conclusions

In this paper, the propagation of thermal waves by an elliptical hole in an infinite block was investigated based on the non-Fourier law of heat conduction. A general solution of the scattering field of thermal waves based on the wave model of heat conduction was presented. The distributions and variations of temperature amplitude under different parameters were analyzed and discussed. The following conclusions can be drawn:

(1) When the wave number was comparatively large, the effects of varying temperature were great. Otherwise, the effect was narrow.

(2) The eccentricity of the ellipse had a great effect on the temperature distribution. When the eccentricity was greater, the temperature drop from the circular case was greater. When the major radius of the ellipse was along the lateral scale, the temperature showed great fluctuation, and along the longitudinal scale, the temperature showed small fluctuation.

(3) The position of the hole in the block was another factor that influenced the temperature distribution. When the center of the hole was on the median of the block, the temperature curve was symmetrical; otherwise, the curve was asymmetrical.

(4) When the scale of the block was small, the maximum temperature at the surface of the materials was great.

(5) The shallower the hole was, the greater the effect of the hole on the temperature.

(6) The thermal wave propagation characteristics showed microparticle properties under higher-frequency heat load.

Furthermore, the elliptical hole is only an example, and if proper mapping functions Ω(ζ) are given, the application range of this method to solving the problem of the scattering of thermal waves can be spread to arbitrarily shaped holes. Of course, our above study also has some limitations. The object in this research was a block with two boundaries, and increasing the number of boundaries would mean that the results from using this theory would not be sufficiently accurate. An experimental verification system should be established in any future studies. The theory and numerical results of this paper can be used for infrared thermal wave imaging, the physical inverse problem, and the evaluation of internal holes in materials.

## Appendix A

The complex function form of the scattering field of thermal waves by non-circular holes:

$$\begin{array}{ll} \vartheta^{\left(s\right)} & =\sum_{n=-\infty }^{\infty }A_{n}H_{n}^{\left(1\right)}\left(\kappa \left|z\right|\right){\left\{\frac{z}{\left|z\right|}\right\}}^{n}+\sum_{n=-\infty }^{\infty }A_{n}\{\sum_{m=1}^{\infty }{\left(-1\right)}^{1}\left[H_{n}^{\left(1\right)}\left(\kappa \left|z-z_{1m}\right|\right){\left\{\frac{z-z_{1m}}{\left|z-z_{1m}\right|}\right\}}^{(-1)n}\right]\\ & +\sum_{m=1}^{\infty }{\left(-1\right)}^{2}\left[H_{n}^{\left(1\right)}\left(\kappa \left|z-z_{2m}\right|\right){\left\{\frac{z-z_{2m}}{\left|z-z_{2m}\right|}\right\}}^{{\left(-1\right)}^{2}n}\right]+\sum_{m=1}^{\infty }{\left(-1\right)}^{3}\left[H_{n}^{\left(1\right)}\left(\kappa \left|z-z_{3m}\right|\right){\left\{\frac{z-z_{3m}}{\left|z-z_{3m}\right|}\right\}}^{{\left(-1\right)}^{3}n}\right]\\ & +\cdots +\sum_{m=1}^{\infty }{\left(-1\right)}^{l}\left[H_{n}^{\left(1\right)}\left(\kappa \left|z-z_{lm}\right|\right){\left\{\frac{z-z_{lm}}{\left|z-z_{lm}\right|}\right\}}^{{\left(-1\right)}^{l}n}\right]\} \end{array}$$

For the convenience of the calculation, we took l=4, and the scattered field of the thermal wave field can be described as:

$$\vartheta^{\left(s\right)}=\sum_{n=-\infty }^{\infty }A_{n}H_{n}^{\left(1\right)}\left(\kappa \left|z\right|\right){\left\{\frac{z}{\left|z\right|}\right\}}^{n}+\sum_{n=-\infty }^{\infty }A_{n}\sum_{l=1}^{4}\sum_{m=1}^{\infty }{\left(-1\right)}^{l}H_{n}^{\left(1\right)}\left(\kappa \left|z-z_{lm}\right|\right){\left\{\frac{z-z_{lm}}{\left|z-z_{lm}\right|}\right\}}^{{\left(-1\right)}^{l}n}$$

where $z_{1m}=i2\left(mL-c_{2}\right)$, $z_{2m}=i2mL$, $z_{3m}=-i2\left[\left(m-1\right)L+c_{2}\right]$, $z_{4m}=-2imL$; $L=c_{1}+c_{2}$; m=1,2,⋯∞; exp(iφ)=Ω(ζ)/|Ω(ζ)|, r=|Ω(ζ)|, z=Ω(ζ), $z^{\prime }=\Omega^{\prime }(\zeta )$.

## Appendix B

The expression of $E_{n}$ and E:

Εn=12κRe[ζz′z¯|ζ|||z||][Hn−1(1)(κ|z|)−Hn+1(1)(κ|z|)]{z|z|}n+niIm[ζz′|ζ|z]Hn(1)(κ|z|){z|z|}n+12κ∑m=1∞∑l=14(−1)lRe[ζz′(z¯−zlm¯)|ζ||z−zlm|][Hn−1(1)(κ|z−zlm|)−Hn+1(1)(κ|z−zlm|)]{z−zlm|z−zlm|}(−1)ln+ni∑m=1∞∑l=14Im[ζz′|ζ|(z−zlm)]Hn(1)(κ|z−zlm|){z−zlm|z−zlm|}(−1)ln

$$Ε=-\vartheta_{0}\left\{q\sin\varphi \cos\left[q\left(c_{2}+y\right)\right]+ip\cos\varphi \sin\left[q\left(c_{2}+y\right)\right]\right\}\exp\left[ip\left(x+b\right)\right]$$

where $z=re^{i\varphi }$, z=Ω(ζ); $\zeta =\rho e^{i\theta }$; cosφ=Rez|z|, sinφ=Imz|z|; $X_{n}=A_{n}$.

## References

[1] Thomas R.L., Favro L.D., Grice K.R. (1982). Thermal wave imaging for nondestructive evaluation. Can. J. Phys..

[2] Qiu T.Q., Tien C.L. (1992). Short-pulse laser heating on metals. Int. J. Heat Mass Transf..

[3] Maillard S., Cadith J., Bouteille P. (2012). Non-destructive testing of forged metallic materials by active infrared thermography. Int. J. Thermophys..

[4] Ping S. (2006). Mesoscopic Phenomena—Introduction to Wave Scattering, Localization, and Mesoscopic Phenomena-10.

[5] Fang X.Q., Liu J.X., Hu C. (2010). Nondestructive evaluation of a conducting sphere in semi-infinite functionally graded materials using thermal wave method. NDT E Int..

[6] Garrido F., Salazar A. (2004). Thermal wave scattering by spheres. J. Appl. Phys..

[7] Tzou D.Y. (1995). A unified field approach for heat conduction from macro-to-micro-scales. J. Heat Transf..

[8] Tang D.W., Araki N. (1996). Non-fourier heat conduction in a finite medium under periodic surface thermal disturbance. Int. J. Heat Mass Transf..

[9] Ma X.B., Ye S.L. (2014). Scattering of thermal waves by a heterogeneous subsurface spheroid inclusion including non-fourier effects. Thermochim. Acta.

[10] Bouteille P., Legros G., Bodnar J.L. (2014). Non-destructive testing of metallic materials using passive, and active infrared thermography. Mech. Ind..

[11] Thibaud J.B., Carminati R., Greffet J.J. (2000). Scattering of a diffusive wave by a subsurface object. J. Appl. Phys..

[12] Amirian E., Dejam M., Chen Z. (2018). Performance forecasting for polymer flooding in heavy oil reservoirs. Fuel.

[13] Saboorian-Jooybari H., Dejam M., Chen Z. (2016). Heavy oil polymer flooding from laboratory core floods to pilot tests and field applications: Half-century studies. J. Pet. Sci. Eng..

[14] Salazar A., Sánchez-Lavega A., Celorrio R. (2003). Scattering of cylindrical thermal waves in fiber composites: In-plane thermal diffusivity. J. Appl. Phys..

[15] Terron J.M., Sanchez-Lavega A., Salazar A. (2000). Multiple scattering effects of thermal waves by two subsurface cylinders. J. Appl. Phys..

[16] Wang F., Ma X.B., Chen D.Z. (2015). Thermal wave scattering in functionally graded materials containing a spherical inclusion. Thermochim. Acta.

[17] Terron J.M., Sanchez-Lavega A., Salazar A. (2001). Multiple scattering of thermal waves by a coated subsurface cylindrical inclusion. J. Appl. Phys..

[18] Ma X.B., Jiang H.Q., Chen D.Z. (2016). Thermal wave scattering by a cylindrical subsurface inclusion in semi-infinite slab. Appl. Therm. Eng..

[19] Muskhelishvili N.I. (1956). Some Basic Problems of the Mathematical Theory of Elasticity.

